# Transition Stages in Adjustment of Wives With Their Husbands’ Erectile Dysfunction

**DOI:** 10.5812/ircmj.16594

**Published:** 2014-03-05

**Authors:** Giti Ozgoli, Saeideh Ziaei, Fazlollah Ahmadi, Mahyar Azar

**Affiliations:** 1 Department of Reproductive Health and Midwifery, Faculty of Medical Sciences, Tarbiat Modares University, Tehran, IR Iran; 2 Nursing Department, Faculty of Medical Sciences, Tarbiat Modares University, Tehran, IR Iran; 3 Department of Psychiatry, Faculty of Medicine, Shahid Beheshti University of Medical Sciences, Tehran, IR Iran

**Keywords:** Erectile Dysfunction, Sexual Behavior, Population Dynamics, Family Characteristics

## Abstract

**Background:**

No study has been conducted yet on the process of adjustment of wives with their husbands’ erectile dysfunction in the transitional stages, and there is lack of understanding of this process in Iran.

**Objectives:**

A qualitative, grounded-theory study was designed to examine the process of adjustment of wives with their husbands’ erectile dysfunction in transitional stages.

**Materials and Methods:**

Purposive sampling was carried out in Tehran, Iran. Data collection occurred until the theoretical saturation was reached. A total of 16 semi structured in-depth interviews were conducted with 15 woman participants. The constant comparative method of data analysis was used.

**Results:**

The women were 29-53 years old and duration of marriage was 2-40 years. They had different educational status ranging from Illiterate to Master’s degree. The present study showed the process of adjustment of wives with husbands’ erectile dysfunction in categories of husband broken role, ups and downs in woman’s sexual life, passing through failure, and end of transition. Following erectile dysfunction (event) and the man’s reaction, broken role occurs (change). In response to this change, reactions due to loss of intimacy occur in the ups and downs of woman’s life. Some women, unable to get through the failure, continue low quality life with sexual and communicational problems (limbo). By the end of transition, some women manage to overcome this unpleasant state of limbo, and begin to experience a new life, with increased intimacy, with or without sexual intercourse (new beginning).

**Conclusions:**

If the process of transitional adjustment occurs in women, it will be effective in improving the relationship and increased intimacy, even sexual intimacy. With this understanding, better counseling and therapeutic interventions can be planned for these couples.

## 1. Background

Erectile dysfunction (ED) is a common problem among men, and according to the definitions it is persistent or recurrent inability of man to achieve or maintain penile erection for pleasurable sexual activity (1). In erectile dysfunction, there is a change in a husband’s role to perform intimate sexual act with his wife. Some events in life such as illness and changes in health status lead to the transition process (2). In fact, transition is people’s response in getting through the change, and how they experience the change and respond to it is divided into different phases (3-5).

Studies indicate the effects of ED on men including reduced self-esteem, self-confidence, sense of masculinity (6), avoiding spouse (7), social isolation, symptoms of depression (8), denial, anger, and acceptance (9). Family as a system naturally has a tendency to balance itself. An unusual event for one of the members always provokes a compensatory response by another member (10, 11). Since sex is a two-way relationship (12), ED affects both man and woman. Thus, depending on behaviors and reactions of the man to this problem, the spouse’s response can be different. Studies reveal that effects of ED on women vary from negative attitudes, dissatisfaction with the problem (13), taking no action, ignoring the problem to persevering men’s sense of masculinity, and relief from intercourse (7) to efforts for interaction and intimacy by women to minimize distress felt by men (7, 14).

Sexuality is a complex, and multi-faceted issue, related to other dimensions of life. As a need, sexuality exacts dynamic changes in life, and as a structure, it is influenced by biological, psychological, social, cultural, and spiritual factors (15, 16). Thus, perception of sexuality varies in different societies. In the review of studies, an understanding of the process of adjustment of wives with their husbands’ ED is not recognized in Iran. Considering the shame in expressing sexual problems, lack of a routine assessment of sexual health, inadequate access to official sexual counseling centers, lack of official sex education, in Iran, sexual dysfunction in the power axis of the family, who do not attend for treatment, will result in reactions by their wives, influenced by this culture. The current study intended to seek a deep understanding through a qualitative study. Thus, grounded theory that has the ability to find the relationship between concepts and meanings through understanding the process of adjustment of wives with their husbands’ ED was used.

## 2. Objectives

A qualitative, grounded-theory study was designed to examine the process of adjustment of wives with their husbands’ erectile dysfunction in transitional stages.

## 3. Materials and Methods

 The study was conducted with a qualitative design and grounded theory approach in Tehran, Iran. This method is used to obtain rich data, and to clarify social processes inherent within human interactions (17). The current study aimed an in-depth examination of experiences of wives with their husbands’ ED in the real world, and to explain their adjustment process. The study began with purposed sampling and continued with theoretical sampling with the most variant samples. This type of sampling, through obtaining diverse information leads to better assessment of the nature and various dimensions of the phenomena (17, 18). Wives of men with at least six months -ED entered the study. This meant that women had the necessary experience of the effects of this problem on their sexuality and adjustment.

In the current study, data were collected through interviews and field notes. Interviews were conducted first by explaining the study objectives and obtaining participants consents. The face to face interviews were conducted semi-structured. The interviewer had passed courses and attended workshops for qualitative research methodology and qualitative interviews and had gained enough experience. All participants were assured of confidentiality of data and that their details would not be revealed in published reports. Before each interview, participants were asked to describe their married life, then questions related to sexuality and factors affecting it were asked. According to the level of participants’ responses, interviews lasted between 20 to 80 minutes each. In every interview, in addition to asking general study questions, depending on the responses, attempts were made to conduct interviews personally. Sampling continued until theoretical saturation. Eventually, 16 interviews were conducted. After the 10th interview, data were almost saturated, but based on the use of grounded theory, interviews continued until the analysis reached theoretical saturation in the 16th interview.

To analyze the data, Strauss and Corbin analysis method and continuous comparison were used. In this method, data collection and analysis were conducted simultaneously (19). The extracted text from each interview was coded. First, data were read line by line and open codes from or close to words used by participants were extracted. The obtained codes were compared to previous ones, and conceptually similar codes were placed in the same category, and gradually, categories were formed. Categories were also compared, and if required, integrated. In some cases, one category was divided into two or more categories, or code of a category was changed to that of another category, and eventually, axial categories were obtained. Selective coding indicated relationship between categories. It should be noted that, with analysis of the interviews, field notes from observations were also coded in the same way.

To ensure the rigor in qualitative research, conformability, dependability, and transferability were used (18). Immersing in data was one of the factors of rigor, and participants’ reviews were also used to confirm integrity of data and codes. This meant that after coding, interview texts were returned to some participants to ensure correctness of codes and their interpretations, and codes that did not reflect participants’ views were modified. The wide range of samples in terms of age, education, intensity of the spouse’s dysfunction, and duration of marriage provided increased credibility of data (20).

Some of the interview texts were reviewed by observers (other than the study team members), therefore, in addition to the research team, extracted codes and categories were also examined by observers, and there was high agreement in the extracted results. To confirm transferability, results were also shared with non-participating women, who approved the suitability of results. The present study was approved by the medical ethics committee of Trabiat Modares University (D52823), and verbal consent was obtained. All the participants were informed about the method and goals of the study. They were informed that participation in the investigation was voluntary and they could stop that at any stage if they wished. Moreover, the participants were reassured that their responses would be confidential and that their personal identity would be protected at all stages of the study.

## 4. Results

**Figure 1.**
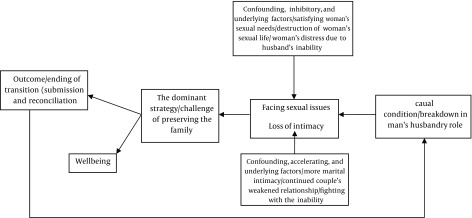
Facing Sexual Issues in Wives of Men with Erectile Dysfunction Straus and Corbin used coding paradigm to connect the events. This paradigm focuses on causal condition, context, intervening condition, consequence and etc. it is used for simplicity of discovering the constructs and the relationship between phenomena, concepts and categories (19).

The outcome of findings showed that a man’s reactions following ED leads to break down of his role as a husband, and under such circumstances the way a woman confronts with sexuality is feeling of loss of sexual intimacy and subsequent emotional losses in her married life. Ups and downs in a woman’s life such as satisfying her sexual needs, destruction of sexual life, and distress with husband’s inability are the inhibitory confounding and underlying factors in this confrontation. Accelerating confounding factors are more marital intimacy continued, weakened marital relationship, and fighting with the inability. As a result and at the end of this transition, a few women effectively talked about sex life, the problem, and how to solve it with their husbands. Another group of women who consider this problem and its consequences as a problem purely related to men adjust their sexual needs to those of their husband’s, suppress it, or ignore it, so they can, with overwhelming strategy of maintaining the family, remain committed to married life without sex, or with bad sex (Figure 1). Table 1 presents personal characteristics of participants in brief. Based on the data, the process of adjustment of wives with their husbands’ ED was identified in categories of broken role, ups and downs in woman’s sexual life, passing through failure, and end of transition (Table 2). 

**Table 1.** Personal Characteristics of Participants

Details	Min-Max
**Age, y**	29-53
**Duration of marriage**	2-40
**Number of children**	0-5
**Education**	Illiterate – Master’s Degree
**Age when married**	11-36
**Number of marriages**	1-2
**Occupation **	Housewife – employed
**Age of the spouse, y**	32-80
**Age difference with spouse**	1 - 36 years older

**Table 2.** Axial Code, Categories and Sub-Categories

Axial Code
**Breakdown in husband role of the man**
Persistence of dysfunction
Influenced and influencing associated
Factors of dysfunction
Response to treatment
Neglecting treatment
Reduced attraction of sexual needs
Man’s selfish attitude
Desperation (man)
Avoiding wife
Lack of commitment
Inattention
Aggression
Denial
Frustration
**Ups and downs in woman’s married life**
Satisfying woman’s sexual needs
Acceptable sexual relationship for woman
Demand to satisfy woman’s sexual need
Sexual awareness
Destruction of sexual life for woman
Unacceptable sexual relationship
Avoiding relationship
Ignoring sexual needs
Woman’s distress due to husband’s inability
Failure
Negative feelings toward husband
Loss of wife’s place
Suspicion about marital commitment
**passing through the failure**
Dealing with inability
Treatment without consulting doctor
Treatment with consulting the doctor
Continued weakened relationship
Disturbed relationship
Detached marital relationship
More marital intimacy
More sexual intimacy
More non-sexual intimacy
**End of transition**
Compatibility and adjustment
Sexual reconciliation
Suppression of sexual needs
Commitment to marriage without sex
Well being
Couple talking about sexual problem
Change in belief and behavior of woman to solve the problem
Active and efficient involvement of woman in seeking treatment

### 4.1. Breakdown in Husband Role of the Man

Intensity of ED in men is followed by inability to have a normal sexual relationship. This dysfunction may be severe with total disability, or partial with sporadic sexual intercourse. Severity of disability can vary depending on factors causing the disorder such as diabetes, addiction, old age, or psychological factors and also men’s response to treatment.

### 4.2. Man’s Inability to Establish a Sexual Relationship

Participant 10: It has been 7-8 years since the problem started, but it is not so bad as to bother me. His problem is occasional. I think it happens when his blood sugar is high (43 years old, spouse 49, married for 32 years). Participant 3: He has been to the doctors, and taking medication for three months now, he is a lot better (27 years old, husband 32, married for two years).

### 4.3. Neglecting Treatment

On the other hand, these men neglect essential treatment; sometimes because of reduced attraction of current sexual life, or due to a selfish attitudes. Participant 14: He doesn’t want to do anything about it. He’s been to doctors, and taken his medicine once, and left the treatment half way through. He doesn’t want to solve his sexual problems, he is lying, he hasn’t been to the doctor’s (30 years old, husband 34, married for six years). Participant 2: He says he couldn’t do it from the start and that’s all-. He’s enjoyed his relationship with his first wife, now it’s not important to him. But, sexual relationship is important to me (44 years old, husband 80, married for eight years).

### 4.4. Desperation (Man)

Following this disorder, man shows reactions to this disability, especially if disability is complete, then he feels he cannot meet his wife’s sexual needs. Thus, he shows reactions like rejection and avoiding his wife, or even aggression, and neglecting, therefore the consequences will be the wife’s not getting close to him, hiding the problem, denial, and even exhaustion, or non-commitment to marital life.

Participant 4: I didn’t know that this sexual problem affected men’s moods, he picks on everything, he is very isolated, runs away from me, and he’s so far away, making the relationship very cold and changes our moods, making us nervous (33 years old, husband 32, married for two years).

Participant 2: Now he is angry when I say I need [sex], he picks a fight (44 years old, husband 80, married for eight years).

Participant 14: It bothers me that he watches porn films so much. Six months ago I told him, he said he is relieved when he watches them. And I was angry and told him; you have a wife. He knows how it hurts me and how much I cry, he still carries on (30 years old, husband 34, married for six years).

### 4.5. Ups and Downs in Woman’s Life

Following previous sexual experiences or their natural need for sexual satisfaction that has been an objective of their marriage, awareness of sexual needs after marriage, or satisfying sexual needs is important for these women.

#### 4.5.1. Satisfying Woman’s Sexual Needs

Participant 1: Before marriage, I didn’t know this feeling at all. My need was stimulated with that of his, we enjoyed it a lot. Now that he has no needs, it is as if I have none, either. I wish our sexual relationship were like before, but he cannot (29 years old, husband 45, married for five years)

Participant 2: My need is stimulated with that of my husband’s. For a month or two, when he used medication, our relationship was good, we did it twice a week, and I was in high spirits. I felt younger. Life is all about this. It’s not so important to my husband because he’s enjoyed himself with his first wife. When we had sex, I was so happy, now I feel I am withered (44 years old, husband 80, married for eight years).

Participant 13: Before this problem, we had sex 2-3 times a week. We both enjoyed it very much. It is so important. I think it strengthens life. A woman can be fully in charge of her life when she does it. If it doesn’t exist, intimacy is lost (38 years old, husband 37, married for 20 years).

Participant 3: It may be due to the mentality that you should enjoy it, and when you don’t you do not feel right. You cannot understand if you have not experienced it. A normal sexual relationship is good and enjoyable. I don’t know about the frustration, what am I supposed to say; it is so bad (27 years old, husband 32, married for five years)

Participant 8: Well, I liked it, I would prepare myself, and used to say, I like to be with my husband. We both enjoyed it. Now I am hurt, it is as if I have no husband. That’s how it is, can’t do anything about it (43 years old, husband 45, married for 23 years)

#### 4.5.2. Destruction of Sexual Life for Woman

Behaviors and reactions of a man, not just because of lack of intimacy due to the erectile dysfunction, but the break down in the man’s role as a husband which follows, causes loss of sexual intimacy, which results in emotional vacuum in a woman’s marital life.

Participant 14: I liked him to be pleased when I made myself up, and understand, but it’s not important to him, he is indifferent. I liked him to hug me, caress me, and look at me with love. When my needs and expectations of affection were not met, I became frigid (30 years old, husband 34, married for six years)

Participant 5: He turns his back to me and sleeps, he cannot understand. He deprives me of a simple kiss and cuddle. I feel I am getting farther and farther away from him, also he from me (33 years old, husband 40, married for 14 years).

Participant 13: He slept on the floor, away from me. Our relationship had become very cold. He had become so sensitive that even if accidently our feet touched, he would pull himself away (38 years old, husband 37, married for 20 years).

#### 4.5.3. Woman’s Distress due to Husband’s Inability

When these women are faced with their husbands’ inappropriate compensatory behaviors and lack of affection and sexual intimacy, in addition to feelings of frustration due to lack of sexual intimacy, they also develop negative feelings toward their husband, and find their feminine place lost, as someone who can satisfy her husbands’ sexual needs, and they may even suspect involvement of other women in their husband’s life.

Participant 16: It is two years now that we have not had sex, we do not even have foreplay, I do not love him at all. I do not think he loves me either (38 years old, husband 41, married for nine years).

Participant 7: When I get close to him, he pushes me away, he says he is tired, and wants to sleep. I feel distraught; I feel he does not even look at me. I feel worthless. Any woman, however ugly and helpless likes to be loved by her husband. But I do not have such a feeling. When he comes to me every other month, I say to myself, maybe he is not that cold yet and still loves me (42 years old, husband 48, married for seven years).

Participant 5: I always say, it is as if I am a servant in this life. I do not think I am a wife at all. I am sorry, I said go with anyone you like. I feel his heart is not with me anymore, and does not like me anymore. He is not satisfied. I feel he is with someone else and enjoys being with her (33 years old, husband 40, married for 14 years).

Participant 1: One feels bad when her husband cannot do anything, it has become mundane. He has less feeling, less affection (29 years old, husband 45, married for five years).

Participant 11: I feel angry when my needs are not met. I hate him (33 years old, husband 60, married for two years).

Participant 4: After 3 months I became suspicious, and said, “don’t you like me anymore?, don’t you fancy me?” (33 years old, husband 32, married for two years).

### 4.6. Passing Through the Frustration

There are different ways of getting through this stage for couples. Sometimes man or even the woman takes action to self-treat ED without consulting the doctor, or swap doctors in the hope of a quick cure for this disorder, or associated problems such as infertility.

#### 4.6.1. Dealing with Inability

Participant 15: My husband has been to the doctors. Every two or three months, he takes a pill, and drinks tea on top, and he is ready in ten minutes (53 years old, husband 70, married for 40 years).

Participant 4: We have tried different doctors, and used any tablets they prescribed, we even tried traditional medicine, and medications advertized on TV, but he was alright for a short time, and then back to square one (34 years old, husband 33, married for two years).

#### 4.6.2. Continued Weakened Relationship

Some people continue with their married life despite their loose relationship, broken marital relationship, lack of sexual intimacy, and its subsequent emotional vacuum.

Participant 16: We have nothing in common to talk about. We have not had sex for two years. He beat me once, and our life even worsened since then. I do not love him at all, and do not think he loves me, either (38 years old, husband 41, married for nine years).

Participant 14: I have no feelings for him anymore. Right now I do not want to even see him. It is a troubled feeling I have toward him. A few days ago he was on a mission, and I was so worried, I did not want him to touch me. When he is around I am very anxious (30 years old, husband 34, married for six years).

#### 4.6.3. More Marital Intimacy

Besides these actions, some people experience having more sexual and non-sexual intimacy without intercourse.

Participant 1: I say it is a good method, let’s try it. Making love without intercourse (29 years old, husband 45, married for five years).

Participant 3: It is not his fault, poor thing. Specially, he has become so kind afterward. The frustration is forgotten (27 years old, husband 32, married for three years).

### 4.7. End of Transition

By the end of this transition, some of these women consider it purely a problem associated with their husband, and adjust their sexual needs to those of their husband’s. Therefore, whenever he could and wanted to, they would be intimate, or if the man was not able to, then the woman will not want to either, and suppresses it, or she ignores it for as long as she can to preserve her family and married life without sex.

#### 4.7.1. Compatibility and Adjustment

Participant 5: There are two options; he is not going to change. Either I have to play away, which I cannot do, because of my life and my children, or I have to kill this need, and keep myself busy with other things. Since he is not goanna change (33 years old, husband 40, married for 14 years).

Participant 12: He says if I did not bother him so much, it would not be like this. Of course, it is not true. It is to do with his diabetes. I do not want to have sex anyway because I have to bring up 3 young boys, which is the most important thing (42 years old, husband 48, married for 23 years).

#### 4.7.2. Well Being

However, only a few of these couples talk about their sex life, in relation to this problem, and how to resolve it.

Participant 1: Now, we talk about this problem. It was not just because of this relationship that I was with him. I think he is ill, and I have to help him. I did not think that he cannot satisfy me, since my love for him is more important than sex. Even if he does not want to, I force him. Sometimes, I go to him, and he shouts, but I would not let go. My pride was at stake, but I insisted, although, it was not as enjoyable as before. With this illness we found things we did not know before. It was like an incentive to get to know each other better (29 years old, husband 45, married for five years).

Participant 13: I took a long look at myself and my behavior, and I realized my appearance had changed so much that my husband did not like, and I did not pay attention to him like before. The psychiatrist gave me some advice. I sacrificed my pride, and took the initiative. I began to look after myself again, lost weight, and paid more attention to my husband. This led to my husband to change a lot. When I changed, he changed. After 12 months we have sexual relationship again (38 years old, husband 37, married for 20 years).

## 5. Discussion

Results showed that in couples’ normal sex life, man’s reaction to ED changes marital relationship. Perceived reactions by wives of men with ED are the cases such as avoiding wife or even aggression and loss of interest, hiding the disorder and denial and exhaustion, or lack of commitment to married life. These effects of ED in men in other studies have been mentioned as reduced libido, feeling of not being loved and not loving (21), condemning and considering sexual partner as the culprit, higher fidelity (22), reduced relationship with sex partner, fear of intimate behavior with spouse and not being able to meet her needs, and thus avoidance (7), social isolation, and symptoms of depression (8), reduced self-esteem and self-confidence (23), reduced sense of masculinity, and less sexual relationship and satisfaction (24). Haddon cites that ED may cause behavioral changes in the patient like avoiding intimacy, and creating tempered mood, which leads to increased anxiety and heightened ED, and thus, creating a vicious circle of increased anxiety and failure and induced reactions (25).

This breakdown in husband’s role due to behaviors and reactions of the man with ED is the end of normal sex life, which leads to reactions in woman such as negative feelings toward husband and loss of her feminine place as someone who can sexually satisfy her husband. The vicious cycle of increased anxiety and failure due to ED occurs to both the patient and his partner in life (25). This action and reaction of couples lead to loss of sexual intimacy and eventually to emotional vacuum in the ups and downs of woman’s sexual life. Others also argue that relationship and emotional problems are the most important perceived sexual problems (26, 27). Relationship and intimacy are more important to women than sexual relationship (7, 28). And non-sexual reward of sexual response in woman is the emotional intimacy, welfare and lack of negative effects due to avoiding sexual intercourse (29) of which the case was deprived.

In fact, inappropriate interpersonal relationship that occurs in ED patients (30) develops both general and sexual relationship problems between some women and their husbands. In getting through these changes (the limbo) some women have to continue marital relationship despite the agonizing instability and even emotional separation, with a low quality of life or sometimes try to solve the problem through inappropriate self-therapy and self-focus methods. Other studies show that there is a relationship between ED , quality of life , unfavorable partnership (31-34), increased communication problems, and reduced intimacy between the couples (35), although solving the communication problem is the most important factor in long term and effective treatment (36), adjustment in communication is the strongest predictor in treatment success of ED (37). Thus, these women are not successful.

Some couples attempt to get through this problem by creating more sexual and non-sexual intimacy. For these women, adjustment to change is completed by the process of transition as a complex and essential mechanism. Therefore, when this belief is changed or created in woman that this is not a problem of affection, but a disease, that in its influence in married life the woman also has an effective role, and in fact the effects of this problem work both ways, then with active participation they can both take steps to solve the problem. In this way, in addition to change of behavior and attitude toward her husband, woman becomes an effective companion and encourages her husband in all stages of diagnosis and treatment. Based on the change in woman’s attitude, a feeling of getting closer, and forming more sexual and emotional intimacy (a new beginning) is created between the couples (whether the erectile problem has been resolved or not). Although woman suffers not having sexual relationship, it is transitional, and the frustration occurs every time with unsuccessful intimate relationship.

Grester et al. (38) in their grounded theory study with the aim of treatment motivation in men with ED and their partners’ role stated that measures taken for treatment motivation included talking to each other, and highlighting the fact that it was a common problem for both partners. Although talking about this subject was unusual for the woman, when she was the initiator of talk and encouraged the man, the result was very promising. This talk reduced feeling of hopelessness and obstacles in man, and reduced woman’s sense of guilt. Woman talking results in increased treatment seeking behavior in the man. It was not just talking, but how to talk was important. Thus, the female partner was advised to initiate talk, to talk about her own feelings, and to say it was a common problem, and to talk when the man was in a good mood and happy.

In fact, although participation of women for more successful and continued treatment of ED has been emphasized in various studies (13, 37-39) clearly, for successful effect of women on treatment, both partners should attend the clinic (1, 13). The problem is that often men attend the clinic alone, and for the few wives that are invited, there are cultural, social, and religious obstacles in their evaluation and in nearly half the men with ED, talking to their sexual partners or consulting a specialist is difficult (40, 41). When men have the incentive through their wives (42), or through information in articles, the media, friends, and counseling, they attend for treatment (8). In a nationwide study, only 10% of men attend treatment sessions (43), and in the ones who attend, even if the ED is resolved through oral medication, it does not necessarily lead to sexual satisfaction, and communicational issues such as feeling of insecurity, anger, and despair remain. Thus, the treatment approach for this disorder is a combined medical and medicinal, psychotherapy, and sex-therapy, with emphasis on cognitive-behavioral change and communication reform between the couples (1, 37). However, unfortunately, most of these couples do not even enter any discussion to resolve the problem, and without any educational or counseling and therapeutic interventions continue married life without sex or with bad sex, which could affect their overall relationship.

 Therefore, although the present study confirms the results of other studies regarding the central role of women in successful treatment of ED, the valuable finding of this study is identifying the process of transition that follows the breakdown of husband’s role. Furthermore, behavioral-cognitive changes found by the woman through her own efforts, help from specialized counseling, and life experiences led to her improved relationship with spouse, so that she could compensate the lost sexual intimacy by having sexual relationship without intercourse. Thus, interventional programs directed from woman toward man at least improve the relationship. This will cause enhanced motivation of men for treatment, increased success rate and continuation of treatment, and satisfaction induced by treatment especially in women. These are recommendations for future studies in Iran that might show positive or negative results.

The present study showed the process of adjustment of wives with their husbands’ ED in categories of breakdown in husbands’ role, ups and downs in women’s sexual life, getting through the failure, and end of transition by considering different phases of transition stages. If the transitional process of adjustment happens through the women, the minimum achievement for women will be improved relationship, and reformed behavior and attitude. The process in women is probably an important step toward encouragement of their husbands for treatment and its continuation, and wives of men with ED can be taught communicational skills, so that they can initiate and continue relationship, and actively listen to their husband, therefore he can express his thoughts and emotions and understand feelings of his wife, and attempt to solve the problem constructively.

The most important limitation in this study was interviewing women only, thus issues were investigated only from women’s perspectives, if there were no obstacles in interviewing men, the results would probably be richer. Lack of definite diagnosis of ED by an urologist or psychiatrist and sufficing to participants’ statements was one of the limitations. However, given the type of study (qualitative), women’s experiences were the criteria.

The strongest point of the study in examining process of adjustment of wives with their husbands’ ED was the attention paid to different phases of transition stages, as was expected from grounded theory design. Another important strong point of the study was having the maximum variance in samples regarding the severity of the ED in their spouses, duration of marriage, and age. Although sexuality is a culturally sensitive issue and people are embarrassed to talk about it; given the communication skills and experiences of the interviewer important information was obtained. These findings especially women’s attitude toward men was still another strong point of the study, which occurred in the cultural context.
